# Ampelopsin attenuates brain aging of D-gal-induced rats through miR-34a-mediated SIRT1/mTOR signal pathway

**DOI:** 10.18632/oncotarget.12811

**Published:** 2016-10-21

**Authors:** Xianjuan Kou, Xingran Liu, Xianbing Chen, Jie Li, Xiaoqi Yang, Jingjing Fan, Yi Yang, Ning Chen

**Affiliations:** ^1^ Hubei Key Laboratory of Sport Training and Monitoring, College of Health Science, Wuhan Sports University, Wuhan, China; ^2^ Graduate School, Wuhan Sports University, Wuhan, China; ^3^ College of Medicine, Hubei University for Nationalities, Enshi, China

**Keywords:** ampelopsin, aging, autophagy, miR-34a, SIRT1-mTOR signal pathway, Gerotarget

## Abstract

The underlying molecular mechanisms for aging-related neurodegenerative diseases such as Alzheimer's disease (AD) are not fully understood. Currently, growing evidences have revealed that microRNAs (miRNAs) are involved in aging and aging-related diseases. The up-regulation of miR-34a has been reported to be associated with aging-related diseases, and thus it should be a promising therapeutic target. Ampelopsin, also called dihydromyricetin (DHM), a natural flavonoid from Chinese herb *Ampelopsis grossedentata*, has been reported to possess multiple pharmacological functions including anti-inflammatory, anti-oxidative and anti-cancer functions. Meanwhile, it has also gained tremendous attention against neurodegenerative diseases as an anti-aging compound. In the present study, the model rats with D-gal-induced brain aging revealed an obvious expression of miR-34a; in contrast, it could be significantly suppressed upon DHM treatment. In addition, target genes associated with miR-34a in the presence of DHM treatment were also explored. DHM supplementation inhibited D-gal-induced apoptosis and rescued impaired autophagy of neurons in hippocampus tissue. Moreover, DHM activated autophagy through up-regulated SIRT1 and down-regulated mTOR signal pathways due to the down-regulated miR-34a. In conclusion, DHM can execute the prevention and treatment of D-gal-induced brain aging by miR-34a-mediated SIRT1-mTOR signal pathway.

## INTRODUCTION

Alzheimer's disease (AD) is the most prevalent aging-associated neurodegenerative disease in the population with over 65 years old. The pathological hallmarks including the deposition of senile plaque and neurofibrillary tangles (NFTs) in AD are due to the loss of hippocampal and cortical neurons as the clinical manifestation of gradually impaired cognitive capacity [1]. Numerous factors including amyloid precursor protein (APP), Tau and beta-site APP cleaving enzyme 1 (BACE1) are involved in the pathogenesis of AD, but the accurate mechanism of AD is still largely unknown, and effective treatments are still lacking. Up to date, the regulation of non-coding RNAs has gained tremendous interest and attention [2].

MicroRNAs (miRNAs) are small non-coding RNAs with 18-25 nucleotides in length, and can negatively regulate mRNA stability and protein expression. miRNAs are abundant in brain and play an important role in neurodevelopment, synaptic plasticity and pathogenesis of neurodegenerative disorders [3, 4]. Increasing evidence suggests that the alteration in miRNA expression could be implicated in the pathogenesis of AD. miR-34a is a critical player for the induction of senescence, cell cycle arrest and apoptosis, which is highly correlated with a variety of aging-related diseases including AD [5]. This finding highlights the fact that miRNAs could be the novel targets for pharmacological interventions.

Autophagy, an evolutionarily conserved process in eukaryotic organisms, is involved in the degradation of long-lived proteins, cytosolic components, or damaged organelles that is essential for maintaining cellular homeostasis [6–8]. However, the functional status of autophagy is involved in the pathogenesis of AD [9, 10]. The deficient autophagy could be considered as one of the primary factors contributing to disease pathogenesis, suggesting that the regulation of autophagy could be a valuable strategy for the prevention and treatment of neurodegenerative diseases. In addition, silent mating type information regulation 2 homolog 1 (sirtuin 1, SIRT1), is an NAD-dependent deacetylase for modulating cellular metabolism, extending lifespan, and delaying the onset of a number of neurodegenerative disorders including AD [11], which is confirmed to be a direct target of miR-34a [12]. SIRT1 also can improve cell tolerance to environmental stress and aging through activating autophagy. Recent studies have demonstrated that miR-34a is involved in p53 and SIRT1 signal pathways [13], thus implying that miR-34a may be involved in the regulation of autophagy.

Up to date, phytochemicals from traditional herbs have gained increasing attention in the field of studies against aging and aging-related diseases. *Ampelopsis grossedentata* is a medicinal and edible plant widely distributed in southern China. Ampelopsin, a natural flavonoid from *A. grossedentata*, also called dihydromyricetin (DHM), has been reported to have anti-inflammatory, antioxidant and anti-tumor functions [14]. DHM has been confirmed to execute its protective function against H_2_O_2_ and 6-OHDA-induced neurotoxicity in PC12 cells by regulating ERK, AKT and GSK-3β/NRF2/ARE signal pathways [15, 16]. These findings suggest that DHM is a promising candidate for the prevention and treatment of neurodegenerative diseases. Moreover, several flavonoids such as quercetin, resveratrol and naringin with similar chemical structures as ampelopsin can prevent or attenuate brain aging induced by D-galactose (D-gal), ameliorate pathological characteristics of AD, and improve cognitive capacity in different aging-related models [17–19]. What's more, recent reports have documented that miRNAs can be regulated by dietary and pharmacological agents [20]. Based on above documentation, DHM could execute possible inhibition on miR-34a and its corresponding targets for the prevention and treatment of D-gal-induced aging were systematically explored.

The mTOR and SIRT1 signal pathways are involved in the regulation of aging through modulating the functional status of autophagy [21]. Since DHM can execute its promising prevention and treatment of aging-associated diseases. Thus, we hypothesize that its potential for prevention and treatment of aging-associated diseases may be accomplished by induced autophagy through miR-34a/SIRT1/mTOR signal pathway. In the present study, the D-gal-induced rat model with brain aging was used to validate our hypothesis through evaluating protein and gene expression associated with miR-34a/SIRT1/mTOR signal pathway, which will provide a new theoretical basis for the application of DHM in the prevention and treatment of aging-associated diseases, and a theoretical reference for the development and utilization of natural products for health promotion and disease therapy.

## RESULTS

### DHM alleviated D-gal-induced learning and memory impairment

**Figure 1:**
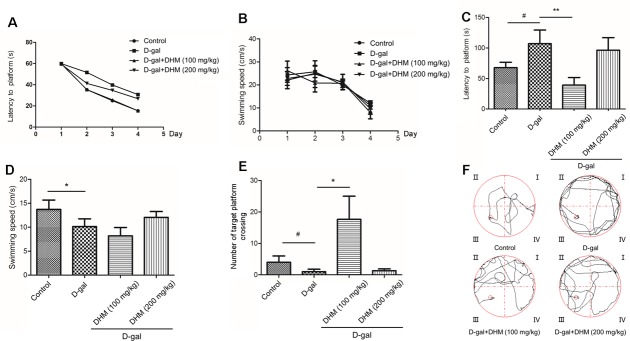
Changes in learning and memory capacity in rats administrated normal control group, D-gal group, D-gal + DHM (100 mg/kg) group, D-gal + DHM (200 mg/kg) group assessed by morris water maze (MWM) (*n* = 10 per group) **A.** Latency to platform of the rats during 1-4 days MWM training. **B.** Swimming speed of the rats during 1-4 days MWM training. **C.** Latency to platform of the rats on the 5^th^ day. **D.** Swimming speed of the rats on the 5^th^ day. **E.** Typical swimming patterns of space exploring. F) Number of target platform crossing. #*p* < 0.05, ##*p* < 0.05 and ###*p* < 0.001 relative to the normal control group; *p < 0.05 and **p < 0.01 relative to the D-gal treatment group.

The spatial learning and memory capacity of D-gal-induced aging rats was evaluated by MWM test. Consistent with previous reports, the escape latency of the rats on the first day of swimming training in the groups except D-gal model group was longer than that on other days and then decreased gradually during 4 days swimming training (Figure 1A). In the MWM task, swimming speeds of the rats from all groups had no difference during 4 days and no difference among three groups (Figure 1B). As shown in Figure 1C, on the 5^th^ day, the escape latency of the rats in D-gal-induced model group was significantly longer than that in the normal control group. However, DHM treatment significantly shortened the escape latency when compared with D-gal-induced model group.

In addition, as shown in Figure 1D, on the fifth day of swimming training, the mean swimming speed of D-gal-induced aging rats was significantly decreased when compared with that in the normal control group, suggesting their aging behavior. However, DHM treatment tended to increase the mean swimming speed although no significant difference in swimming speed between DHM and D-gal model groups was observed. As shown in Figure 1E and 1F, the number of crossing the platform position in D-gal model group revealed a significant decrease when compared with the normal control group, indicating the impairment of learning and memory capacity. As we expected, DHM treatment markedly increased the number of crossing platform position when compared with D-gal model group and normal control group. Therefore, DHM can significantly improve learning and memory capacity.

### DHM mitigated the damage or senescence of hippocampal neurons in D-gal-induced rats

**Figure 2:**
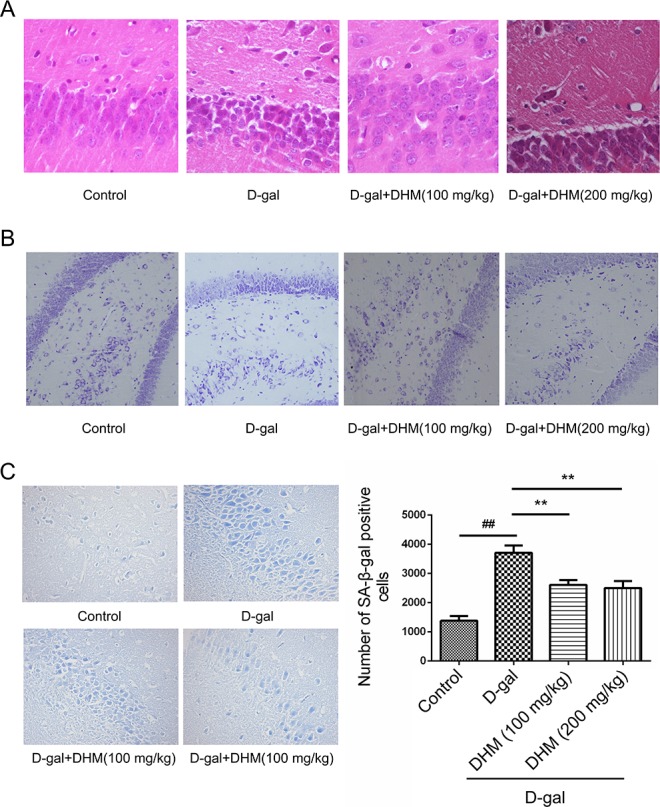
**Representative photomicrographs showing histopathological changes in hippocampus tissues with A.** HE (scale bar, 50 μm), **B.** Nissl staining (scale bar, 100 μm) and **C.** SA-β-gal staining (200×), #*p* < 0.05, ##*p* < 0.05 and ###*p* < 0.001 relative to the normal control group; *p < 0.05 and **p < 0.01 relative to the D-gal treatment group.

In order to investigate the damage or senescence of hippocampal neurons in D-gal-induced rats, histopathological changes were examined by HE and Nissl staining. HE staining showed significantly increased cell death of neurons in the CA3 region of hippocampal tissue from D-gal-induced aging rats. In contrast, the rats subjected to DHM treatment exhibited a significant decrease in damaged hippocampal neurons, suggesting that DHM can effectively attenuate the injury of hippocampus tissue induced by D-gal in aging rats (Figure 2A). Based on Nissl staining, normal cellular viability of hippocampal neurons was observed in the normal control and DHM treatment groups. Compared with the normal control group, the population of neurons was significantly decreased in D-gal model group; moreover, the number of Nissl bodies in D-gal model group was significantly decreased and neurons were damaged or lost in the CA3 region of hippocampus tissue (Figure 2B).

SA-β-gal staining was used to evaluate the senescence of the cells induced by D-gal. D-gal could induce a significant increase of senescent cells with positive staining (blue color) for SA-β-gal when compared with the normal control group; in contrast, DHM pretreatment at the daily dose of 100 or 200 mg/kg markedly reduce the population of positive cells stained by SA-β-gal (Figure 2C).

### DHM down-regulated D-gal-induced miR-34a in hippocampus tissue

**Figure 3:**
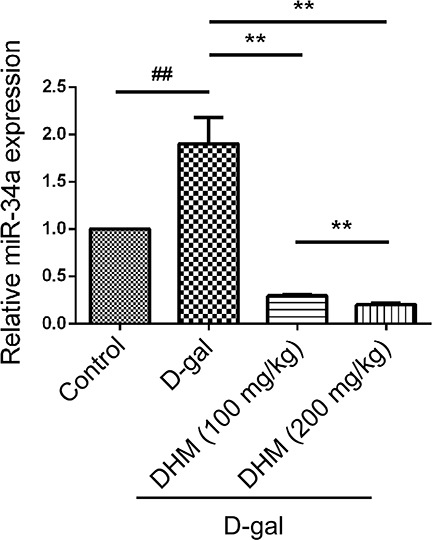
The miR-34a was activated in hippocampus tissue in D-gal-induced aging rats The expression of miR-34a in different groups was analyzed through quantitative RT-PCR.

We examined the change of miR-34a expression by qRT-PCR through comparative Ct (ΔΔCt) method in hippocampus tissue before and after DHM treatment. The expression of miR-34a in hippocampus tissue was distinctly different in each group. As shown in Figure 3, D-gal treatment significantly increased the expression of miR-34a when compared with the normal control group. However, the administration of DHM markedly mitigated the up-regulation of miR-34a induced by D-gal. These results revealed that the up-regulation of miR-34a was positively correlated with brain aging of D-gal-induced rats.

### DHM ameliorated D-gal-induced aging of hippocampal neurons through up-regulating SIRT1 and down-regulating p53/p21

**Figure 4:**
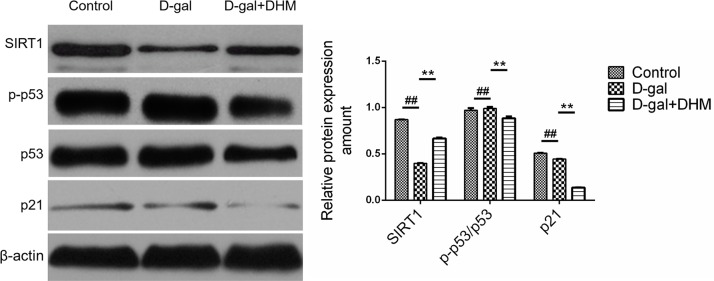
The effect of DHM on SIRT1/p53 signaling in hippocampus tissue of D-gal-induced aging rats SIRT1 and p-p53, p53 and p21 were evaluated by Western blot. The data were expressed as mean ± standard deviation (M ± SD) from independent experiments performed in triplicate. Equal protein loading was confirmed by β-actin.

As a tumor suppressor, p53 is a key factor caused by aging. Many factors inducing senescence can activate p53 signal pathway and promote cellular senescence [22]. In order to verify whether miR-34a-induced senescence is correlated with p53, we determined the protein expression of p-p53, p53 and p21 by Western blot. As shown in Figure 4, a positive correlation among p53, p21 and miR-34a was observed. However, DHM supplementation could markedly alleviate this change. Among them, the expression of p-p53 and p21 in rats subjected to the daily administration of DHM at 100 or 200 mg/kg revealed a little difference, so 100 mg/kg as the daily dose of DHM for subsequent experimental evaluation was chosen.

In order to examine whether down-regulated miR-34a by DHM is connected to the modulation of SIRT1 signaling, the effect of DHM on SIRT1 protein expression was also evaluated using Western blotting analysis. As shown in Figure 4, D-gal could cause a significant decrease in the expression of SIRT1 protein. On the contrary, SIRT1 was significantly increased by DHM and displayed a 2-3-fold enhancement when compared with that in D-gal model group. These results suggest that DHM can result in the phosphorylation of SIRT1 and p53.

### DHM suppressed D-gal-induced apoptosis and rescued dysfunctional autophagy

**Figure 5:**
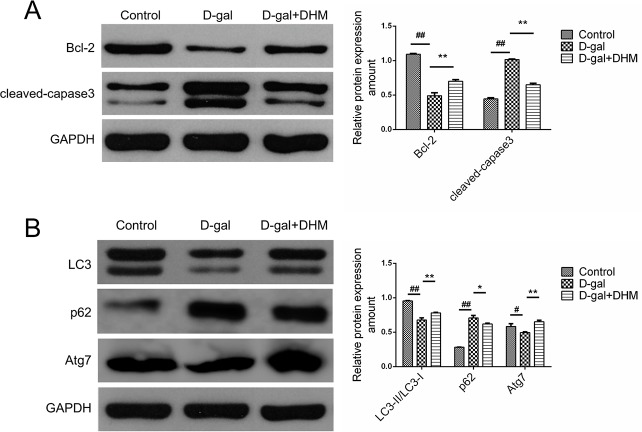
DHM attenuated the excessive apoptosis and rescued the deficient autophagy in hippocampus tissues of D-gal-induced aging rats **A.** Apoptosis-associated proteins such as Bcl-2 and Caspase-3 were subjected to immunoblot analysis using corresponding antibodies. **B.** Autophagy-associated proteins such as LC3, p62 and Atg7 were analyzed by Western blot using corresponding antibodies. The data were expressed as mean ± standard deviation (M ± SD) from independent experiments performed in triplicate. Equal protein loading was confirmed by GAPDH.

In order to explore the regulatory role of DHM in functional status of apoptosis or autophagy, we examined the effect of DHM on the expression of pro-apoptotic protein caspase-3 and anti-apoptosis protein Bcl-2 using Western blot. As shown in Figure 5A, D-gal administration induced an obvious increase in cleaved caspase-3 and a significant decrease in Bcl-2 in hippocampus tissues of D-gal-induced aging rats when compared with the normal control group. However, compared with the D-gal model group, DHM treatment significantly decreased the expression of cleaved caspase-3 and up-regulated Bcl-2, suggesting that DHM can exert an anti-apoptotic function in D-gal-induced cell damage.

As shown in Figure 5B, the protein expression of Atg7 and LC3-II/LC3-I ratio in D-gal model group was dramatically decreased, suggesting the D-gal-induced dysfunctional autophagy when compared with the normal control group. However, pre-treatment with DHM significantly rescued the reduction of autophagy-associated markers. As the substrate of autophagy, p62 regulated the formation of protein aggregates. Consistent with above results, hippocampus tissues from the rats in D-gal model group showed increased p62 level, which was attenuated by DHM supplementation.

### DHM attenuated D-gal-induced astrocyte activation in hippocampus tissue

**Figure 6:**
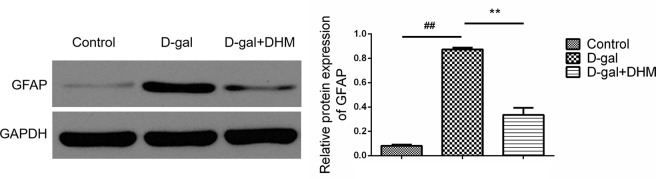
DHM reduced the overexpression of GFAP in hippocampus tissue of D-gal-induced aging rats The data were expressed as mean ± standard deviation (M ± SD) from independent experiments performed in triplicate. Equal protein loading was confirmed by GAPDH.

The injury of central nervous system is commonly accompanied by astrocyte activation and reactive gliosis, and characterized by an increase in GFAP [23]. As shown in Figure 6, the expression of GFAP is significantly increased in hippocampus tissues of the rats from D-gal model group; however, the treatment with DHM could result in a significant decrease of GFAP, suggesting the attenuation role of DHM in astrocyte activation.

### DHM inhibited mTOR signal pathway in hippocampus tissue of D-gal-induced aging rats

**Figure 7:**
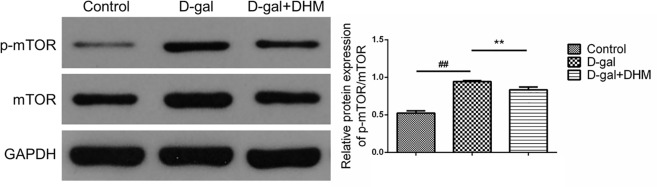
DHM inhibited mTOR activation in hippocampus tissue of D-gal-induced aging rats through the evaluation by Western blot using p-mTOR and mTOR antibodies The data were expressed as mean ± standard deviation (M ± SD) from independent experiments performed in triplicate. Equal protein loading was confirmed by GAPDH.

mTOR is a negative regulator of autophagy. Therefore, autophagy can be activated through pharmacological or genetic inhibition of mTOR. In order to determine whether mTOR accounts for DHM-mediated protective effect, we examined mTOR activation in D-gal-induced aging rats by Western blot. As shown in Figure 7, interestingly, DHM supplementation significantly reversed the increased phosphorylation of mTOR at Ser^2448^ (p-mTOR) during D-gal administration, which suggests that DHM could activate autophagy through inhibiting mTOR signaling.

## DISCUSSION

According to previous reports, DHM shows neuroprotective effects on *in vitro* models of Alzheimer's disease, Parkinson's disease and nerve injury [15, 16]. However, evidences *in vivo* and in human beings are still lacking. Thus, we have further investigated the pharmacological effects of DHM on neurodegenerative diseases in animal models. Natural aging rat is the best animal model for the investigation of brain aging. However, the exploration of aging-associated diseases using natural aging rat model is still limited due to the difficult availability of natural aging rats, high aging-associated mortality, susceptibility to cancers, and other complications such as hypertension and diabetes, which could result in the high impact on the reliability of experimental results. Since chronic D-gal-induced aging model has been proposed and confirmed to induce learning and memory impairment due to neurodegeneration and lipid peroxidation [24], we have applied D-gal-induced aging rat model with mimic characteristics of natural brain aging process to explore the prevention and treatment of aging-associated diseases and underlying molecular mechanisms. In the current study, D-gal administration resulted in accelerated aging and impaired spatial learning and memory, excessive apoptosis and dysfunctional autophagy of hippocampal neurons, and astrocyte activation associated with aging when compared with the normal control group. In contrast, DHM supplementation improved learning and memory capacity, suppressed cell apoptosis and astrocyte activation, and activated autophagy in hippocampus tissue of aging rats when compared with those aging-associated indicators in the D-gal model group.

Accumulating miRNAs have also been considered as the critical modulators of cellular senescence. Recently, emerging evidences have shown that miR-34a is over-expressed in aging-related diseases including AD [25]. Based on these findings, we have analyzed the expression of miR-34a in D-gal-induced brain aging model. Consistent with previous reports [5], our data have also shown that increased miR-34a in D-gal-induced aging model is involved in common pathological events of brain aging. Interestingly, DHM treatment can significantly decrease gene expression of miR-34a, which stimulates us to further explore the target genes of miR-34a during the rescuing process of brain aging upon DHM treatment. It is well known that p53 and p21 are involved in the regulation of cell cycle, while SIRT1 is a negative regulator of cell senescence and can mitigate several aging-associated diseases [26]. In our study, D-gal-induced overexpression of senescence-associated proteins including p-p53, p53 and p21 and down-regulation of SIRT1 in hippocampus can be recused upon DHM supplementation with the accompany of a significant decrease in miR-34a expression. Thus, DHM-induced up-regulation of SIRT1 protein may compensate the reduction of aging-associated genes or proteins. However, the underlying mechanisms of activating SIRT1 by DHM still need to be further explored.

Oxidative stress can lead to neuron apoptosis, which is involved in neurodegenerative diseases. As shown in Figure 5A, cell apoptosis is markedly increased in hippocampus tissue of D-gal-induced model rats; in contrast, DHM administration can attenuate D-gal-induced apoptosis. Therefore, miR-34a may represent not only an initiator of apoptotic signal pathway, but also a promising therapeutic target in preventing the death of neurons during aging process. In addition, Bcl-2 has also been described as the target of miR-34a [27], so we have evaluated its protein level to reveal a significant decrease in D-gal-induced aging rats and an obvious up-regulation upon DHM administration, suggesting the expression of miR-34a is highly correlated with the aging and DHM can accomplish its anti-aging function through inhibiting apoptotic signal pathway under the down-regulated miR-34a condition.

Recent studies have also demonstrated that the activation of astrocytes accompanying with Aβ deposition in the brain of AD model mice [28] and in AD human brain [29] is characterized by an increase of GFAP. Consistent with previous results, learning and memory dysfunction caused by D-gal is combined with the increased GFAP as an indicator of astrocyte activation in hippocampus, suggesting that astrocytes respond to aging-related cognitive impairment. Interestingly, DHM treatment can effectively inhibit GFAP in hippocampus of D-gal-induced rats to execute its neuroprotective effect.

miRNAs can interact with many cellular signal pathways through silencing mRNA, thereby providing new therapeutic means to alter the dramatic evolution of cognitive signs detected in aging and aging-related diseases. Autophagy, as a catabolic pathway, can be involved in the degradation of cellular components *via* the lysosomal machinery to maintain health neurons by eliminating damaged organelles and protein aggregates [7]. However, impaired or defective autophagy can lead to the accumulation of damaged protein and intracellular organelles, and even promote cell death. A decline in autophagic function is a common trait of aging process. Numerous evidence has implicated defective autophagy in the pathogenesis of several major neurodegenerative diseases, particularly AD [30, 31], thereby, elevating the functional status of autophagy could have therapeutic potential.

In order to determine function status of autophagy, we have evaluated the autophagic activity through determining LC3 and p62 by Western blot. In D-gal model group, the markedly increased p62 level and down-regulated LC3 and Atg7 in hippocampal neurons were observed, which suggests that the deficiency of autophagy in hippocampus of the D-gal-induced aging rats should be the partial mechanism of brain aging and impaired learning and memory. However, the deficient autophagy in hippocampus from D-gal-induced aging rats can be ameliorated upon DHM supplementation.

At present, it is generally believed that autophagy and mTOR-mediated autophagy signal pathways are at least partly responsible for aging and a variety of aging-related neurodegenerative disorders [32]. Activating mTOR complex can inhibit autophagy. As we expected that DHM can deactivate mTOR signal pathway in D-gal-induced aging rat model. In addition to maintain energy homeostasis, SIRT1 also can regulate autophagic degradation. According to previous reports [33], resveratrol and many polyphenolic compounds can improve the activity of SIRT1 *in vitro,* thereby stimulating SIRT2 activity and extending the lifespan by approximately 70% in yeast with mimic role of caloric restriction. Thus, small molecular compounds of SIRT1 activators could have therapeutic potential in several aging-related diseases. Moreover, SIRT1 can deacetylate several components in the complexes during the formation of autophagosomes, such as Atg7 and Atg5 proteins [34]. Because SIRT1 can regulate different targets in autophagic pathway, and be activated under different conditions, SIRT1 regulation could be more important during aging process. Consistent with these observations, we have observed that DHM can improve SIRT1 activity, thereby increasing Atg7 protein level. What's more, DHM has multiple cellular targets like other natural compounds. Some studies have demonstrated that p53 is a repressor of autophagy [35]. Studies on the aging models have revealed that p53 signal pathway undergoes the interaction with SIRT1 as well as autophagic regulation, suggesting that SIRT1 is also able to regulate autophagic degradation through p53 signal pathway. Consistent with above reports [36], our findings have revealed that DHM treatment can significantly inhibit p-p53 and p53, thus correspondingly inducing autophagy in D-gal-induced brain aging model.

In conclusion, DHM can ameliorate cognitive impairments in D-gal-induced aging rats by the induction of autophagy in hippocampus tissues through suppressing aging-related astrocyte activation and inhibiting mTOR signal pathway as well as down-regulating miR-34a, which provides the important theoretical supports of DHM for preventing or treating aging-associated neurological disorders.

## MATERIALS AND METHODS

### Drugs and reagents

Ampelopsin (CAS No. 27200-12-0) was purchased from Zelang Medical Technological Co. Ltd (Nanjing, China). It was purified from natural products by high performance liquid chromatography (HPLC) with the purity of more than 98%. D-gal was ordered from Sigma-Aldrich Corporation (St. Louis, MO, USA). The senescence β-galactosidase staining kit was purchased from Cell Signal Technology (Danvers, MA, USA). Primary antibodies including Bcl-2, Bax, LC-3, p62, SIRT1, phosphor-p53, p53, p21, β-actin and GAPDH were purchased from Cell Signaling Technology (Danvers, MA, USA). All secondary antibodies for Western blot were purchased from Cell Signaling Technology (Danvers, MA, USA).

### Animal grouping and treatments

Totally 40 male Sprague-Dawley (SD) rats (age: 8 weeks old; body weight: 160 ± 20 g; certification No.: SCXK(e)2015-0018) were obtained from the Experimental Animal Center of Hubei Provincial Disease Control Center (Wuhan, China). The protocols were reviewed and approved by Institutional Animal Care and Use Committee at Wuhan Sports University. The rats were randomly divided into four groups including normal control group, D-gal model group, and D-gal combined with DHM at the doses of 100 and 200 mg/kg-d groups with 10 rats in each group.

All rats were housed at the environment with room temperature of 22 ± 2°C and a dark-light cycle (12 h: 12h), and provided the accessibility to food and water ad libitum. After adapting to new environment for 1 week, the rats from DHM groups were administered with DHM dissolved in distilled water at the designated dosages by gavage once a day at 8:00am for 6 consecutive weeks. The rats from the normal control group were administrated with distilled water. Except from the normal control group, the rats from other groups were subjected to subcutaneous injection of D-gal at the dose of 150 mg/kg.d for 6 consecutive weeks. Each administration of DHM should be 2 h ahead of D-gal injection.

### Behavioral testing

After DHM treatment for 5 weeks, morris water maze (MWM) procedure was used to evaluate hippocampus-dependent spatial learning and memory capacity of the D-gal-induced aging rats. The MWM is consisted of a circular pool (150 cm in diameter) filled with water. A platform was submerged 2 cm under water in one of four identical quadrants. The MWM task lasted 5 days. The first day represented the positioning test. Rats were released into water at different starting points and subjected to four trials each day to find the platform. Rats failed to find the platform within 60 s were gently guided to the platform and allowed to stay on the platform for 15 s. The escape latency that rats spent in finding the platform and the swimming speed were recorded. On the fifth day, rats underwent the space-exploring test in which the platform was removed. Rats were allowed to swim freely for 60 s. The latency to the platform (the time for finding the original platform area), the time spent in the target quadrant and the adjacent quadrants (left quadrant, right quadrant and opposite quadrant), the number of target platform crossing, and the swimming speed were recorded.

### Senescence SA-β-galactosidase (SA-β-gal) staining

SA-β-gal staining was used to evaluate cell senescence. Hippocampus tissues from the rats in different groups were harvested, fixed and stained using an SA-β-gal staining kit (Sigma, St. Louis, MO, USA) according to the manufacturer's instructions. The number of cells with positive β-galactosidase staining in blue color was counted under an inverted microscope. Three regions with more than 200 cells were randomly selected and the positive cells with β-galactosidase staining in each region were recorded and the average number of cells with positive β-galactosidase staining in three regions was calculated.

### Real-time PCR of miR-34a

Total RNA was extracted from hippocampus tissues of D-gal-induced aging rats using a Takara MiniBEST Universal RNA Extraction Kit (Takara, Dalian, China) and treated with DNase I (Ambion, Austin, TX, USA) to obtain DNA-free RNA according to the manufacturer's instructions, and quantified with a NanoDrop2000 spectrophotometer (NanoDrop Technologies, Wilmington, DE). Reverse transcription of RNA into cDNA was conducted using themiScript Reverse Transcription Kit (QIAGEN, Valencia, CA, USA). Real-time RT-PCR was carried out with SYBR Premix ExTaqTM (Tli RNaseH plus) (Takara, Dalian, China) using an Applied Bio-Rad CFX96 Sequence Detection system (Applied Biosystems, USA), and all reactions were repeated three times. Primer sequences used for PCR amplification of miR-34a included the forward primer of 5'-TGCGCTGGCAGTGTCTTAGCTG-3' and reverse primer of 5'-CCAGTGCAGGGTCCGAGGTATT-3'. The expression level of miR-34a was defined from the threshold cycle (Ct), and relative expression level of miR-34a was calculated using the 2^−ΔΔCt^ method after normalization with reference to the expression of U6 small nuclear RNA.

### Preparation and histological examination of hippocampus and cortex tissues

After MWM task, 5 rats were decapitated under anesthesia. Brain was immediately taken out, and cortex and hippocampal tissues were isolated and frozen in liquid nitrogen for future Western blot. In addition, 5 rats were anesthetized and perfused with 0.9% saline followed by 4% paraformaldehyde (pH 7.4). Brain was removed and immersed in 4% paraformaldehyde at 4°C overnight, and then subjected to sequential paraffin embedding and sectioning at a thickness of 4 μm. Meanwhile, the samples of hippocampus tissue were harvested at the volume of 1 mm^3^, fixed in phosphate buffer containing 2.5% glutaraldehyde for 2 h, then rinsed with 1 mmol/L phosphoric acid solution and fixed in 1% osmium tetroxide for 2-3 h. The block was cut carefully into ultrathin sections at the thickness of approximately of 70 nm. The sections were stained with 3% uranyl acetate and lead citrate and then examined under a JEOL JEM1400 electron microscope at Wuhan Institute of Virology, Chinese Academy of Sciences.

### Western blot analysis

Hippocampus and cortex tissue samples were homogenized in lysis buffer containing 20 mM Tris (pH 7.5), 135 mM NaCl, 2 mM EDTA, 2 mM DTT, 25 mM β-glycerophosphate, 2 mM sodium pyrophosphate, 10% glycerol, 1% Triton X-100, 1 mM sodium orthovanadate, 10 mM NaF, 10 μg/mL aprotinin, 10 μg/mL leupeptin, and 1 mM PMSF for 30 min on ice and centrifuged at 12000 ×g at 4°C for 30 min. The supernatant was collected and protein quantification was carried out using a BCA kit (Walterson Biotechnology Inc., Beijing, China). The protein samples were boiled in the presence of sample buffer at 95°C for 5 min. The target protein was separated by sodium dodecyl sulfate polyacrylamide gel electrophoresis (SDS-PAGE), transferred to nitrocellulose membrane, and then probed by corresponding primary and secondary antibodies. Finally, the target protein was visualized by enhanced chemiluminescence (ECL) reagent exposure to X-ray film.

### Statistical analysis

All data were expressed as mean ± standard deviation (M ± SD). Statistical analysis for single comparison was conducted by Student's *t*-test and the statistically significant difference was considered at *p* < 0.05.
